# The Use of Rose Bengal Plate Test to Asses Cattle Exposure to Brucella Infection in Traditional and Smallholder Dairy Production Systems of Tanga Region of Tanzania

**DOI:** 10.4061/2010/837950

**Published:** 2010-09-29

**Authors:** Emanuel Senyael Swai, Luuk Schoonman

**Affiliations:** ^1^Veterinary Investigation Centre (VIC), P.O. Box 1068, Arusha, Tanzania; ^2^Tanga Dairy Trust (TADAT), P.O. Box 1720, Tanga, Tanzania

## Abstract

A cross-sectional epidemiological study was conducted to determine the seroprevalence and to identify risk factors for bovine brucellosis seropositivity in traditional and smallholder dairy cattle production systems in the Tanga region of North-eastern Tanzania. The study populations comprised 246 indigenous and 409 crossbred cattle, randomly selected from 105 smallholder dairy and 25 traditional managed herds, respectively. Individual animal and herd-level data were collected using a structured questionnaire. Serum samples were screened for *Brucella* antibodies using the Rose Bengal Plate Test The overall seroprevalence of *Brucella* antibodies in the smallholder dairy and traditional managed cattle was 4.1% and 7.3% respectively. The corresponding overall herd prevalence was 10.5% and 20% respectively. Using multivariate logistic regression analysis, closeness to stock route, access to surface drinking water and location were identified as the major risk factors for individual herd seroprevalence. Older animals (≥6 years) were associated with increased risk of sero-positivity compared to animals of age category of ≤6 years. The results showed that brucellosis is prevalent and widely distributed locally, underscoring the need for further studies including surveillance and institution of preventive and control measures particularly among female young-stock and the general public who are at high risk of contracting brucellosis.

## 1. Introduction

Tanzania has a wide variety of livestock production systems influenced by climate, feed availability, culture, and farming systems [1]. Smallholder farmers in pastoral and agropastoral production systems account for about 99% of the total livestock population and support the livelihoods of approximately 80% of the population [2]. Under these production systems, livestock grazing range from communal, extensive grazing to stall fed or tethered grazing on smallholdings [3]. Pastoral livestock production system is dominated by indigenous traditional herds whereas agro-pastoralism comprises a range of combination of low-scale crop cultivation and improved or graded livestock rearing. Smallholder dairy farming is concentrated in the high-potential rural areas (highlands) and in many urban and periurban areas of major cities where milk marketing opportunities is high. The predominant production system in Tanga region is the traditional livestock keeping and small-scale dairy production [2].

Brucellosis is considered by the Food and Agriculture Organisation (FAO), the World Health Organisation (WHO), and the Office International des Epizooties (OIE) as one of the most widespread zoonoses in the world [4]. In cattle, the disease is usually caused by *Brucella abortus* (a gram-negative, facultative intracellular coccobacilli bacterium) and occasionally by *B. melitensis* and *B. suis.* Brucellosis is characterised by late term abortion; infertility and reduced milk production as a result of retained placenta, endometritis, and a varying degree of sterility in the males and cows [5].

The history of brucellosis in Tanzania began in 1927 when an outbreak of abortion in cows was reported in Arusha region [6, 7]. Surveys have shown the disease to occur in cattle in various production systems, regions, and zones, with seroprevalence varying considerably [8–11]. The majority of these studies, which were often conducted purposely, were carried out in parastate farms and in indigenous traditional cattle herds. Surveys carried out in the Eastern zone and in the dairy sector in Tanzania revealed prevalence ranges from 2.2% in small scale (1–10 animals) to 7.6% in large-scale (>30 animals) and 12.3% in pastoral sector, respectively [8, 9]. Reports from individual dairy cattle in Northern Tanzania showed low prevalence (<4%) compared to traditional cattle (15%) in the same zone [6]. In Tanga region, the disease has been insufficiently investigated and information relating to its magnitude, distribution, and risk factors is scant. Such information is important when designing appropriate strategies that would help reduce its prevalence and effects. The present study was carried out in order to gather additional information likely to contribute to data that may be used to devise appropriate national strategies for the control of the disease.

The objective of the serological survey was to determine the prevalence of bovine brucellosis and to identify the associated risk factors under traditional and smallholder dairy production system in the Tanga region, North-eastern Tanzania.

## 2. Materials and Methods

### 2.1. Area and Study Population

The study was conducted on both smallholder dairy herds (≤10 graded animals of all ages, breed, and sex and intensively managed) and traditional herds (≥30 indigenous cattle of all ages and sex and extensively managed) in and around Tanga municipality. Characteristics of the study area are described elsewhere [12, 13]. The location of each herd was also classified as urban (within the official boundary of Tanga municipality), periurban (beyond the official boundary of Tanga municipality but within 15 km of the town centre), or rural and grazing system (grazing versus no-grazing). Such classification of herds by location and grazing system could influence, for example, availability of veterinary input supplies, extension services, affecting access to land for forage production, veterinary input suppliers, or animal health service provisions. Herd classifications (by grazing and location) were considered as explanatory variables during data analysis. The type of animals kept under smallholder herds includes *taurine *breed (Friesian, Ayrshire, Jersey, Simmental) and crosses of these breeds with *Bos indicus* breeds (Tanzania shorthorn zebu, boran, and Sahiwal). The level of *taurine *blood varies from 50%–85%. Traditional herds comprise mainly Tanzania short horn zebu (TSHZ).

### 2.2. Study Design and Farms Selection

A cross-sectional study was undertaken between May 2003 and January 2004. The data bases of farmers under the district livestock department and Tanga dairy development programme [14] were used as the sampling frame. A sampling frame of 1,730 smallholder dairy and traditional herds, comprising 12,500 cattle, was used to select 130 herds to participate in the study. Owing to the practical consideration of logistics and funds, the sampling frame was limited to herds within a radius of 40 km around Tanga municipality. 

Simple random sampling using the Excel software (Microsoft Inc., 1999) was used to select the 130 herds which resulted in the recruitment of 105 smallholder dairy and 25 traditional herds. The sample size of herds and animals was selected to estimate the disease prevalence and association between outcome and explanatory variables at 90 percent confidence, with 7 percent precision based on formulae given by Noordhuizen et al. [15]. 

### 2.3. Questionnaire Design and Data Collection

Information about each herd and the animals kept was collected by means of a structured questionnaire, which was completed at all the selected herds on a single visit. The questionnaire was designed to comprise mostly closed ended (categorical) questions to ease data processing, minimize variation, and improve precision of responses [16]. The questionnaire was administered using the national Swahili dialect by a veterinary department staff member, who was trained in participatory research methodologies. Important herd and animal level data recorded included cattle location, source of forages (home established, road side, or bought-in hay), sex, breeding method used (natural or artificial insemination), source of drinking water (tap, rain water, shallow well, river, pond), contact with other animals and place (between farms, during grazing, at water source, during mating), herd owner education level (binary variable: illiterate and primary education), and the number of years in livestock farming. Other information sought included presence of other animals on the farm (pigs, sheep, goat), history of abortion and S.19 vaccination, disposal of afterbirth, age determined from birth records and dentition characteristics, categorized as (binary variable; ≥6 and ≤6 years), herd-size (binary variable; large ≥9 and low ≤9 animals), and the type of floor in the animal house (concrete, dirt) as well as whether or not a system of grazing or zero-grazing was practiced.

### 2.4. Sample Collection and Handling

Approximately 10 mL of blood sample was collected from the jugular vein of each animal in all selected herds using plain vacutainer tube (Becton Dickson, UK). Each sample was labelled using codes describing the specific animal and herd. The tube was set tilted on a table over night at a room temperature to allow clotting. Next morning, the clotted blood in the tubes was centrifuged (at 3000 g for 20 min ) to obtain clear serum. The obtained serum was stored at −20°C until tested by Rose Bengal Plate Test (RBPT).

### 2.5. Rose Bengal Plate Test

All sera samples were screened using RBPT antigen (VLA Weybridge, UK). The test procedure recommended by Alton et al. [17] was followed. Briefly, 30 *μ*L of RBPT antigen and 30 *μ*L of the test serum were placed alongside on the plate, and then mixed thoroughly. The plate was shaken for 4 min  and the degree of agglutination reactions was recorded. The sample was classified positive if any agglutination was observed and negative if no agglutination. The RBPT, when compared to complement fixation test (CFT), has shown a sensitivity of 94.2% and a specificity of 87% on field sera and has been described by other researchers [18, 19]. Confirmation of positives samples with tests of higher sensitivities and specificities such as a CFT or enzyme-linked immunosorbent assay (ELISA) was not done due to the lack of resources (funds) to buy the required kits.

### 2.6. Statistical Analysis

Herds and individual animal-derived data were stored in Microsoft Access. Descriptive statistics for the animal and herd level explanatory variables examined in the study were developed using Epi-Info version 6.04 d [20] and Statistix version 8.0. [21]. Relationships between explanatory (independent) variables (herd and animal-level) and outcome variables (*Brucella *serostatus: negative or positive) were investigated in two steps by logistic regression. In the first step, relationships between each independent and outcome variable were individually investigated. In the second step, any variables that were significantly associated at the *P* < .10 level were included in multivariable models producing, by forwards and backwards substitution and elimination, the most parsimonious models in which all independent variables remained significant at the *P* < .05 level.

## 3. Results

### 3.1. Participating Herds Characteristics

All 130 of the selected herds were visited and the farmers were interviewed, resulting in a 100% response rate for participation in the study. Smallholder dairy herds made up 80.7% of the herds sampled and traditional herds 19.3%. Samples were collected from 655 animals of which 7% were males and 93% females. The average age of the animals in the traditional sample was 5.7 years compared to 4.6 years in the smallholder dairy sample (*P* < .001). The distribution of herds amongst categories of each variable investigated is summarised in Table 1. All traditional herds were grazing animals whereas the majority (72%) of the smallholder dairy herds was kept under zero-grazing conditions. Smallholder dairy herds had an average herd size of six animals, with larger herds being kept under grazing than under zero-grazing conditions (*P* < .001). Traditional herds had an average herd size of 46 animals (median 30). Goats and sheep were significantly kept more commonly in traditional herds than in smallholder dairy herds (*P* < .001). Traditional livestock keepers keep cattle for longer than small-holder farmers, with an average of 19.1 (median 14) compared with 9.4 (median 9) years, respectively. Forty-percent (*n* = 42) of the smallholders dairy farmers had more than primary education compared to 12% in the traditional sector. Two-thirds (66%) of the smallholder dairy farmers had at least received training in cattle management through workshop or seminars whereas traditional farmers learned cattle management alone or from parents (<0.001). No history of S.19 vaccination was recorded during sampling. Abortion history was recorded in 12% and 3.7% of the herds in traditional and smallholders dairies, respectively.

### 3.2. Serological Responses to Brucella Infection

**Figure 1:**
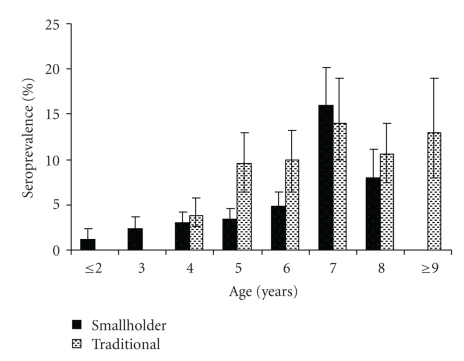
Age seroprevalence profile (+/−95% *C* *I*) of *Brucella* in the smallholder dairy (black) and traditional (stippled) cattle production systems of Tanga (May 2003–January 2004).

Of the 655 sampled animals, serology results were available from 654 (99.8%) animals. The missing result arose due to loss of labels during storage and transport to laboratories. Over all, animal seroprevalence for *Brucella *antibodies was 5.3% (95% Confidence interval [CI], 3.1–7.8). The animal and herd-level seroprevalence of antibodies to* Brucella *by production system are shown in Table 2. The corresponding overall herd seroprevalence (at least one animal or herd seropositive) for *Brucella *antibodies was 12% (95% CI, 10.1–13.9). The relationship between age and seroprevalence is shown in Figure 1. In the traditional herds, the prevalence of cattle seropositive for *Brucella *antibodies increased from 4% in animals <4 year-old to 14% in cattle >6 years of age, while in smallholder dairy herds, the prevalence of cattle seropositive for *Brucella *antibodies increased from 2% in young stock <2 years of age to 16% in stock of >6 years.

## 4. Factors Associated with Seropositivity to Brucellosis

### 4.1. Univariable Analysis

The association of the animal and herd-level categorical explanatory variables at *P* ≤ .10 and brucellosis is shown in Table 3. Animal-level categorical variables that qualified at *P* ≤ .10 during univariable analysis were age and the introduction of animals during the last two years prior to the current study. Herd-level variables that were significant at *P* ≤ .10 during univariable analysis included grazing, herd location, access to surface water, and closeness to cattle stock route or holding ground.

### 4.2. Multivariable Analysis

Explanatory variables that remained significant for the seropositivity to brucellosis, defined by a sero-positive animal and/or herd in the final multivariable regression model are shown in Table 4. The likelihood that a herd was sero-positive for *Brucella* increased significantly for being near to the slaughter cattle stock route (*P* < .001). Herds that were accessed to surface water were significantly associated with increased risk compared to the herds which depended on piped water (OR = 2.66, *P* < .001). Seroprevalence to *Brucella* varied significantly amongst herd locations, with herds located in urban areas being significantly less likely to be sero-positive compared to rural or periurban located herds (OR = 0.37, *P* < .001 for urban). At an animal-level, older animals (≥6 years) were more likely to be seropositive compared to animals ≤6 years old (OR = 4.0, *P* < .001). Seroprevalence varied significantly amongst herd source of drinking water and grazing system but these were strongly confounding significant factors with access to surface drinking water providing the minimal best model fit (Likelihood ratio statistic = 14.1, *P* = .049) in the multivariate analysis. Results of Fisher's Exact Test showed that history of previous abortion (*P* > .05) and stillbirths (*P* > .05) in the individual animals were not significantly associated with *Brucella* seropositivity. None of the other investigated variables were associated with differences in prevalence values.

## 5. Discussion

The overall animal seroprevalence of brucellosis in this study was 5.3%. The seroprevalence could reflect a past or present exposure to *Brucella* organisms, because vaccination against brucellosis has never been practiced in Tanga region, during the last 10 years [14]. The prevalence was higher in animals in traditional herds than smallholder dairy herds, being 7.3% and 4.1%, respectively. Also, at herd level, brucellosis was more prevalent in traditional herds, with 20% showing at least one positive animal. Although animal prevalence is low, the high herd prevalence, especially in traditional herds, shows that brucellosis is common and locally widespread. 

The low prevalence of brucellosis observed in the smallholder dairy animals when compared to traditional herds agrees with the observations made in other studies [8, 22, 23]. The difference is also likely to be explained by the small-size units and stall feeding that minimises contacts between herds and animals. However, the high proportion of seropositive animals in traditional herds, despite differences between herds, conformed to the results of a recent study in Tanzania which also reported higher prevalence of brucellosis in indigenous TSHZ cattle than in crossbred kept by smallholder dairy farmers [11]. The differences between traditional and crossbred animals are possibly attributed to increased contacts of infected herds/animals and noninfected ones in the indigenous traditional production system, as a result of communal grazing and watering, which become more apparent and acute during the dry period. Moreover, mixed herding and frequent contact with small ruminants and cattle could also be contributing factors to the occurrence of brucellosis.

At herd level, farming close to the stock route and access to surface water emerge as the most important risk factors. Slaughter cattle from outside the Tanga region are trekked along the stock route to be slaughtered at the abattoir. Animals are kept for some days before slaughter at a holding ground where they partly make use of the same communal grazing areas. The brucellosis prevalence in these animals is high [24] and direct, or indirect, contact via pasture exposes grazing animals to diseases carried by these animals. Such practices enhance the exposure potential, especially following abortions, through increased contact and common grazing field and watering points promoting transmission of *Brucella* organism [25]. 

The relevant data from East and Central Africa, and from other environments similar to those found in Tanzania, also indicate that *Brucella* antibodies can be quite widespread among the cattle production systems. For example, in the pastoral animal and herds RBPT-based investigations, seroprevalences of 0.77% and 46.1%, 9.3% and 31%, 14.1% and 46.2% have been reported in Ethiopia, Ghana, and Zambia, respectively, [26–29]. The apparent geographical variation in the seroprevalence may reflect differences in the levels of natural immunity, management and husbandry practices employed, and sensitivities and specificities of the diagnostic methods used among researchers as well as genetic variation in disease resistance among the breeds maintained in the systems [27, 30].

In both production systems, the seroprevalence for *Brucella *antibodies increased with age, consistent with previous reports [31, 32]; younger animals are more resistant to primary infection and frequently clear infections, although latent infection occur [33]. The increased likelihood of *Brucella *force of infection in older animals (≥6 years) could be explained by increased susceptibility to infection due to under nutritional stress combined by the lowered immunity that developed following acute infection [5, 28]. 

Compared to traditional herds, animals in the smallholder dairy herds become positive at a slightly younger age. It has been described for brucellosis that some of the infected animals, do not become sero-positive until pregnancy [33]. This could partly explain this difference as the age at first calving for crossbred dairy cattle is lower than for traditional zebu cattle [34].

A history of previous abortions or stillbirths was not significantly associated with seropositivity, a finding which is in contrast with reports from other researchers [24, 26]. Fifteen percent of the RBPT positive animals reported a history of abortion, compared to 9.7% of the RBPT-negative animals. This could be explained by the fact that causes of abortion such as vector and nonvector borne diseases which are prevalent in the study area were important factors rather than brucellosis [13, 35]. Similar observations were made by other investigators [5, 36]. Although RBPT is generally accepted and accredited by OIE [37] as the definite test for *Brucella *screening in many countries, the test has several limitations [38]. Apart from being highly sensitive especially in vaccinated animals, the test has the major drawback of producing false-negative reactors. False-negatives are usually obtained during early stages of incubation or immediate after abortion whereas false-positives occur due to the presence of IgM as a result of S.19 vaccination and colostral antibodies in young stock. The lower specificity is the reason that normally RBPT-positive animals are either confirmed with the complement fixation test (CFT) or enzyme-linked immunosorbent assay (ELISA). 

The study further revealed that sex did not show significant association with the seroprevalence for *Brucella *antibodies. The absence of association with the seroprevalence is in agreement with that of other workers [39] and possibly due to the small sample size of the male cattle sampled.

The possible sources of bias in this study include the fact that herds fell into aggregates with large spaces between them. Furthermore, several of the brucellosis positive herds showed clear clustering. This might have lead to unreliable estimates of seroprevalence. This was overcome during study designing stage by incorporating random sampling procedures that would give a 90% to 95% probability of detection of a disease in a herd with a minimum intraherd prevalence of 10% [40]. Sampling an equal number of herds from each production system that had large sampling frame differences might also have introduced bias. The sampled herds in smallholder dairy production represented 9.3% (409/4375) of the eligible population while in traditional indigenous cattle production it was 3% (246/8125). This was overcome by random selection. Another possible source of bias in this study is the problem of some variables influencing and being associated with *Brucella *seropositivity. Confounding was addressed by using multivariable model. However, other confounders may potentially have resulted in residual confounding. These include grazing and animal access to surface drinking water.

## 6. Conclusion

This study reports that bovine brucellosis is prevalent and widely distributed locally. Seroprevalence was higher in traditional than smallholder dairy herds suggesting traditional herds to be at a higher risk of brucellosis, most likely due to the commingling with small ruminants and frequent contact at communal grazing and watering points. Animals were more likely to be seropositive if they were in traditional herds, accessed to surface running water and graze close to a trade/ slaughter animal stock route. This may reflect the increased likelihood of contacts with contaminated pasture and water from aborted materials, although history of abortions was not a significant risk factor. Consistent with this, the likelihood of encounter increased significantly and logically with age. Further studies are needed to understand the dynamics of transmission cycles and institution of preventive and control measures particularly among female young-stock, and to identify alternative management practices to replace those that are risk factors for animal and human infections.

## Figures and Tables

**Table 1:** The proportions of herd in each category of each variable investigated during the study (May 2003–January 2004).

Variable	Categories	No. of herds (%)	

		Traditional (*n* = 25)	Smallholder dairy (*n* = 105)
Sex†	Male	22 (9)	25 (6)
	Female	223 (91)	384 (94)
Brought in animals from previous years	Yes	5 (21)	29 (27.6)
	No	20 (79)	76 (72.4)
Grazing history	Zero grazing	0 (0)	76 (72.4)
	Semi/free grazing	25 (100)	29 (27.6)
Herd location	Periurban	5 (20)	37 (35.2)
	Urban	5 (20)	53 (50.5)
	Rural	15 (60)	15 (14.3)
Breeding system*	Own bull	13 (52)	13 (12.4)
	Bull from outside	12 (48)	60 (57.7)
	AI	0 (0)	67 (63.8)
Water source*	Tap	4 (16)	87 (82.8)
	Rain water	19 (76)	35 (33.3)
	Shallow wells	1 (4)	11 (10.5)
	River	18 (72)	16 (15.2)
	Pond	11 (44)	23 (22.0)
History of abortion	Yes	3 (12)	4 (3.7)
	No	22 (88)	105 (96.3)
Disposal of afterbirth	Yes	8 (32)	95 (90.5)
	No	17 (68)	10 (9.5)
Cattle going to water source	Yes	25 (100)	23 (22.0)
	No	0 (0)	82 (78.0)
Education level	Illiterate	22 (88)	63 (60)
	Above primary	3 (12)	42 (40)
Other training: livestock keeping	Yes	4 (16)	70 (66.6)
	No	21 (84)	35 (33.3)
Source of fodder*	Road side	Na	75 (71.4)
	Own established	Na	27 (25.7)
	Bought in hay	Na	12 (11.4)
Contact with other animals*	Dairy to dairy	0 ( 0)	86 (82)
	Dairy to zebu	17 (68)	19 (18)
	Contact sheep/goat	22 (88)	0 (0)
	Contact pigs	0 (0)	25 (23.8)
	Contact game	9 (36)	2 (1.90029
Place of contact*	Pasture	23 (92)	29 (27.6)
	Watering point	24 (96)	26 (24.8)
	Dip	6 (24)	22 (20.9)
	Mating	1 (4)	28 (26.6)
	Housing (shoats)	17 (68)	17 (16.2)

*Proportion do not add up to 100% each category was treated as a binary variable; AI: Artificial insemination.

Na: not applicable;

†Sample size (traditional, *n* = 246; smallholder, *n *= 409).

**Table 2:** The prevalence (with exact ±95% confidence intervals) of animal and herd-level seropositivity for *Brucella *by production system (May 2003–January 2004).

	Herd-level		Animal-level	
Production system	Number positives	Seroprevalence, % (± 95% CI)	Number positives	Seroprevalence,% (± 95%CI)
Traditional	5	20.0 (13.8–26.2)	18	7.3 (3.1–12.0)
Smallholder dairy	11	10.5 (8.2–11.8)	17	4.1 (1.9–7.6)
Overall	16	12.3 (10.1–13.9)	35	5.3 (3.1–7.8)

CI: lower and upper limits for 95 percent confidence interval of the seroprevalence.

**Table 3:** Association of antibody to *Brucella *positives and explanatory variables (*p* ≤ .10) in univariable regression models (May 2003–January 2004).

		Animal-level		Herd-level	
Variable	Category	OR	*P*-value	OR	*P*-value
Grazing	No	1.00		1.00	
	Yes	1.69	.09	2.65	.08
Herd location	Periurban + rural	1.00		1.00	
	Urban	0.27	.01	0.37	.10
Introduction of cattle during the last year	No	1.00		Na	
	Yes	1.69	.05	Na	Na
Access to surface water	No	1.00		1.00	
	Yes	2.26	.03	2.79	.06
Herd close to stock route/holding ground	≥1 km	1.00		1.00	
	≤1 km	6.74	<.001	3.22	.03

Na: not applicable; OR: Odd ratio.

**Table 4:** Significant factors associated with seropositivity to *Brucella* infection in multivariable logistic models (May 2003–January 2004).

Variable	Category	OR	95% CI OR	*P*-value
Place of farming				
	Periurban + rural	1.00		
	Urban	0.37	0.13–1.12	<.001
Age of animals				
	≤6 years	1.00		
	≥6 years	4.02	1.86–8.69	<.001
Herd close to stock route/h/ground				
	≤1 km	1.00		
	≥1 km	3.09	1.04–9.17	<.001
Access to surface water				
	No	1.00		
	Yes	2.66	1.90–7.87	<.001

OR: odds ratio for categorical/binary variables; CI OR: lower and upper limits for 95 percent confidence interval of the odd ratio.
